# Level of Knowledge, Attitude, and Practice of Pregnant Women on Antenatal Care in Amatere Health Center, Massawa, Eritrea: A Cross-Sectional Study, 2019

**DOI:** 10.1155/2023/1912187

**Published:** 2023-01-24

**Authors:** Hailemichael Gebremariam, Berhe Tesfai, Seltene Tewelde, Yonas Kiflemariam, Fitsum Kibreab

**Affiliations:** ^1^Ministry of Health, Dekemhare Hospital, Zoba Debub, Dekemhare, Eritrea; ^2^Ministry of Health, Northern Red Sea Zone, Massawa Hospital, Massawa, Eritrea; ^3^Ministry of Health, Hazhaz Referral Hospital, Zoba Maekel, Asmara, Eritrea; ^4^Ministry of Health, Mendefera Zonal Referral Hospital, Zoba Debub, Asmara, Eritrea; ^5^Ministry of Health, Health Research and Resources Center Division, Asmara, Eritrea

## Abstract

**Background:**

Proper antenatal care is one of great means of reducing maternal and child morbidity and mortality. However, determining level of knowledge and practice is vital, and the objective of this study was to evaluate this gap among pregnant women in Amatere Health Center, Massawa city, Eritrea, 2019.

**Methods:**

A cross-sectional study with systematic sampling was conducted. All pregnant mothers who were resident of Massawa city and visiting Amatere Health Center for their current pregnancy were included in the study. An interviewer-administered structured questionnaire was used as data collection tool. Results were presented using descriptive statistics, percent, and frequencies.

**Results:**

A total of 289 pregnant mothers were enrolled in the study with a mean age of 27.7 years. Most mothers reported that high blood pressure (92.4%), maternal smoking (97.6%), alcohol consumption (97.2%), infection (92.7%), and medicines (98.3%) had affected fetal growth during pregnancy. Practically, two-thirds (59.4%) of the mothers were visiting the health facility during the first three months of their pregnancy. Majority of mothers had good knowledge (84.1%) and attitude (99%), but they had low level of practice (45%). Marital status, occupation, gravidity, and parity had showed statistically significant association to their comprehensive knowledge (*p* < 0.001). And their gravidity (*p* < 0.003) and parity (*p* < 0.001) had also showed statistically significant association to their level of practice.

**Conclusion:**

Even though majority of the pregnant mothers had high level of knowledge and attitude, their practice towards ANC was relatively low. Age, marital status, and occupation showed statistically significant association to their comprehensive knowledge. Moreover, multiparous and multigravida mothers were having higher level of knowledge and practice on antenatal care. Enhancing community awareness on early starting of antenatal care and improving their practice through proper counseling are highly recommended.

## 1. Introduction

Antenatal care (ANC) that includes education, screening, counseling, treatment, promoting, and monitoring is an important step for the safety of the mother and fetus [1, 2]. ANC provides both psychological and medical needs of pregnant women within the context of health care delivery system, culture, and religion in which the women live [3]. Regular use of antenatal care by pregnant women gives opportunities to health workers to predict and manage pregnancy complications to ensure acceptable maternal and perinatal outcomes [4].

Confidential inquiries into maternal deaths in developing countries have found a positive association with inadequate antenatal care as a risk factor for maternal mortality [5]. Understanding maternal knowledge and practices of the community regarding care during pregnancy and delivery is required for program implementation [6]. Almost 90% of maternal deaths occur in developing countries, and over half a million women die each year due to pregnancy and childbirth-related causes [7].

According to the Eritrean Population and Health Survey (EPHS 2010), almost all health facilities in the country provide free ANC service to pregnant women. This survey found that 80% of urban and 47% of rural women with a birth in the five years preceding the survey made four or more antenatal care visits for their last birth [8]. Based on a study conducted in North Western Asmara, Eritrea, in 2017, findings revealed that most of the participants had good knowledge related to timing of first ANC visit and participants showed positive attitudes towards the ANC services provided to them [9].

Another study in Eritrea in 2013 revealed that 93% of mothers had attended ANC, at least once, for the most recent pregnancy. The percentage of mothers who checked their pregnancy was pretty high in all zobas which was above 90% except for Southern Red Sea at 83% [10].

It is known that proper antenatal care is one of great means of reducing maternal and child morbidity and mortality. And appropriate level of KAP is critical on maternal and neonatal outcome. This is not well determined in our country in general and in the study site in particular. This gap will be filled by this study and initiate further larger studies.

To the knowledge of the researchers, there are no similar studies done in the study area. Therefore, this study is believed to give the present image of KAP on ANC in Amatere Health Center, which may help to initiate further studies. Thus, the objective of this study was to evaluate the level of knowledge, attitude, and practice of pregnant women on ANC in Amatere Health Center, Massawa city.

## 2. Materials and Methods

### 2.1. Study Setting

This was conducted in Amatere Health Center of Massawa city in all pregnant mothers who were resident of the city and visited the health center for their current pregnancy. The study was done in pregnant mothers rather than all reproductive age group in the community to fill the gap that the level of KAP was not determined before in the study site.

### 2.2. Study Site and Population

This study was conducted in Amatere Health Center, located in Massawa, Northern Red Sea region. Massawa is located about 110 km from Asmara in northeast direction. There are seven health facilities in this subzone: one hospital, one health center (Amatere Health Center), and five health stations. Amatere Health Center is one of the sites for ANC in Massawa city (*Massawa subzone*, *Ministry of Health*, *2019*). So the research was conducted in this site because it gives ANC services more than the other health stations.

### 2.3. Inclusion and Exclusion Criteria

Pregnant mothers who were resident of Massawa city for the past 6 months and who were visiting Amatere Health Center for their current pregnancy were part of the research. Pregnant mothers who live outside Massawa city, mothers who repeated ANC follow-up for the same pregnancy, and mothers who have difficulties to conduct the interview were excluded from the study.

### 2.4. Study Design and Sampling

The study was a descriptive cross-sectional. Study samples were taken based on systematic sampling after estimating the number of women who came to Amatere Health Center during the study period. The estimated number of women was 1053, and the study subjects were 301; then, the sample size was selected every *k*th interval (*k* = 3), so every third women were selected for the study. The first eligible woman who attends the ANC in the first day of the data collection period was interviewed; then, every third woman was enrolled thereafter until the required sample size was obtained. It was conducted on consecutive days until the sample size was completed.

### 2.5. Sample Size Determination

The total population of Massawa city is estimated about 26,339, and around 5267 mothers were in the reproductive age group [11] Expected pregnant mothers in the city were about 1053. Therefore, the sample size for this study was calculated based on various aspects. For the purpose of sample size estimation, since the prevalence of KAP was not known, the present study assumed the prevalence as 50%, confidence level as 95%, and margin of error (*d*) as 5%. The sample size was determined using Daniel's formula. The prevalence as 50% was assumed as there was no similar research in the country in general and in the study area in particular.

(1) $$\begin{array}{c} n=\frac{\left[{\left(z/2\right)}^{2}p\left(1-p\right)\right]}{d^{2}}, \end{array}$$

where *n* is the number of sample, *n* = [(1.96)2(0.5)(1 − 0.5)]/(0.05)2, *z* = 1.96, *d* is the margin of error = 0.05, and *n*1 = 384.

The initial sample size was adjusted to account the size of the population where the expected pregnant mother in Massawa city is 1053 (HMIS).

(2) $$\begin{array}{c} n2=\frac{\text{Ni}}{\left(1+\text{ni}/n\right)},\\ nf=\frac{384}{\left(1+384/1053\right)}=281. \end{array}$$

Considering 10% of nonresponse rate, the final sample size is 313.

### 2.6. Data Collection and Analysis Procedures

Data was collected by using interviewer-administered structured questionnaire. The questionnaire which was developed in English language was translated into local language (Tigrigna) for facilitation of communication between the data collectors and pregnant mothers. The questionnaire includes four sections with sociodemographic details of respondents, history of previous pregnancy, and different questions about knowledge, attitude, and practice. Data were checked for completeness and consistency and corrected accordingly. Then, the data was coded and entered in to CSPro. Once data entry was 100% verified, it was exported to SPSS version 21 for analysis. Chi-squared test was used to assess the association between knowledge, attitude, and practice and the sociodemographic factors. *p* value < 0.05 was considered as statistically significant.

### 2.7. Data Quality and Validity

The quality of data was assured by pretesting the questionnaire, training data collectors, and checking collected data on daily basis by principal investigators. The tool was modified and finalized according to the suggestion and recommendations of the research team. A pilot study on about 10% was conducted four months before starting the research in the study site, and these were excluded from the study sample. The questionnaire was tested and revised based on the pilot study results.

### 2.8. Operational Definitions

Knowledge was assessed about ANC visits, tetanus immunization, investigations, nutritional factors, danger signs of pregnancy, contraception, and personal habits. Each parameter was awarded 1 mark for the correct answer and 0 mark if the answer was wrong. Those who scored 70% and above were considered as having adequate knowledge, and those who scored below 70% were considered inadequate knowledge. Similar approach was used to assess the level of attitude and practice of the mothers.

### 2.9. Ethical Consideration

Ethical clearance to obtain an official permission to conduct this study was requested from Ministry of Health Research and Ethical Clearance Committee on January 24, 2019. Mothers were interviewed after a written informed consent was obtained. Participant's confidentiality was secured, and their information was coded to protect individual data. The mothers had the right to withdraw at any stage of the interview, and they were assured that they will not have any harm to them and their pregnancy by participating in this research.

## 3. Results

### 3.1. Social Demographic Characteristics of Pregnant Women in Amatere Health Center, Massawa, Eritrea

A total of 289 pregnant mothers were enrolled in the study. Majority (59.2%) of them were aged between 20 and 29 years with a mean age of 27.74 (95% CI: 27.13-28.34). Most of them were married (93.8%) with a mean age at marriage of 19.08 (95% CI: 18.49-19.68). Their ethnic proportion was Tigre (52.2%), followed by Tigrigna (24.2%) and Saho (10.4%). About half of the mothers (57.1%) had reached primary and middle levels of education, and they were dominated by Muslims (75.1%) and housewives (77.2%). About 20.8% and 22.2% of the study participants were primigravid and nulliparous, and their mean age at first child was 20.42 (95% CI = 19.58-21.26) (Table 1).

### 3.2. Knowledge of Pregnant Women on Antenatal Care in Amatere Health Center, Massawa, Eritrea

Majority of the mothers (70.2%) reported that they delivered in health facility during their previous pregnancy. Besides, 7.6% delivered at home due to transport problem (6.9%) and no nearby health facility (2.1%). Majority (65.4%) of the mothers answered that the first antenatal checkup should be started in the first three months of pregnancy, and most (95.8%) thought that pregnant woman needs to come for at least four antenatal checkups throughout her pregnancy. Most mothers correctly answered that pregnant mothers need to do blood screening for HIV, screening for blood group, and blood pressure examination during her pregnancy. Majority of the mothers reported that high blood pressure (92.4%), maternal smoking (97.6%), alcohol consumption (97.2%), infection (92.7%), and medicines (98.3%) can affect fetal growth during pregnancy. Almost all (99%) of the mothers said that pregnant woman should deliver her baby in health facility, and 99.7% reported that they should report to health facility when they have the danger signs of pregnancy (Table 2).

### 3.3. Attitude of Pregnant Mothers on Antenatal Care in Amatere Health Center, Massawa, Eritrea

Most mothers (93.4%) strongly agree that antenatal checkup is necessary and screening of infections like HIV (90.3%) should be carried out during antenatal checkup. They were also strongly agreed that blood pressure (90.3%), ultrasound (91.7%) checkups, and dietary habit change (91.7%) are good for her pregnancy. Majority of the mothers had strongly disagreed that home delivery was better than hospital delivery (82%), and smoking (83%) and alcohol consumption (82.7%) does not cause harm to baby (Table 3).

### 3.4. Practice of Pregnant Mothers on Antenatal Care in Amatere Health Center, Massawa, Eritrea

Two-thirds (59.4%) of the mothers were visiting the health facility during the first three months of their pregnancy. About half (43.5%) of them have visited five times, and 16.2% were not vaccinated for tetanus (TT) during their current pregnancy. Majority of them were making changes on their diet (86.9%) and were taking iron and folic acid tablets (88.7%). About 2.1% and 2.4% of the mothers were smokers and consuming alcohol during their pregnancy, respectively (Table 4).

### 3.5. Association between Background of Pregnant Women and Level of Knowledge and Attitude in Amatere Health Center, Massawa, Eritrea

Majority of the mothers had good comprehensive knowledge (84.1%) and attitude (99%). The age and ethnicity of the respondents showed significant association to their comprehensive knowledge. In addition, marital status, occupation, gravidity, and parity had showed statistically significant association to their comprehensive knowledge on antenatal care (*p* < 0.001). The single and unemployed mothers were having poor level of knowledge when compared to the married and housewife mothers (*p* < 0.001). Multigravida and multiparous mothers had good knowledge compared to the primigravid and nulliparous mothers (*p* < 0.001) (Table 5).

### 3.6. Association between Background Characteristics and Comprehensive Practice of Pregnant Women in Amatere Health Center, 2019

The level of gravid and parity of the mothers showed statistically significant association to their level of practice (*p* < 0.003 and <0.001), respectively (Table 6).

## 4. Discussion

Understanding maternal knowledge and practices regarding care during pregnancy and delivery is required for making appropriate interventions. Regular and early booking of antenatal care is vital to prevent the neonatal and maternal complication. Antenatal care coverage in Eritrea is very high, ranging from 84.2% in rural areas to 97.3% in urban areas [12]. The objective of this study was to determine the level of knowledge, attitude, and practice of the pregnant mothers on antenatal care.

This study displayed that the mean age of the mother at first delivery was 20.42 years and 6.9% of the mothers were aged below 20 years with the lowest age at marriage was 13 years. This was similar to other studies that the most common age at first delivery was between 18 and 24 years old and 7.4% were between the ages of 16 and 19 years old when they gave their last birth [13]. This showed that underage marriage is seldom practiced in the community which could further predispose mothers to different maternal and neonatal complications.

The antenatal attendance of at least one visit was 100%, and 96.8% of them reported regular antenatal visits for checkup, which was similar to other study in Eritrea that 96.1% of the study participants had at least one visit and regularity in attendance (70.0%), but this was higher to the national average of 89% [12]. This higher antenatal attendance could be due to that the mothers were from Massawa city which is relatively urban, with higher level of knowledge of mothers mostly influenced by mass media and health professionals.

Two-thirds of the mothers (70.2%) reported that they delivered in health facility during their previous pregnancy and 7.6% delivered at home. The mentioned constraints for home delivery were transport problem (6.9%) and no nearby health facility (2.1%). This was almost similar to the national level for other cities outside Asmara that the 2010 Eritrean Population and Health Survey revealed a great discrepancy in the rate of facility delivery within urban centers, from 92.5% in the capital, Asmara, to an average of 62.5% in other towns [12].

The results of health facility delivery were also almost similar to other study that 74.8% had health facility delivery [14]. The most common reason mentioned for home delivery was lack of transportation (46%) [13]. This shows that even though the rate of hospital delivery is high, the identified limitations can also be solved by construction of waiting homes, which was introduced in most parts of the country by the Ministry of Health, and increasing awareness of the community on the importance of antenatal care and upgrading the traditional birth attendants to skilled birth attendants.

About 2.1% of the pregnant mothers strongly agreed that smoking does not cause any harm to the fetus and they practiced smoking during their current pregnancy. And 2.4% of the mothers were also consuming alcohol during their pregnancy. This showed that their lower knowledge on the potential harmful practices can cause fetal and maternal complications.

This study revealed that 59.4% of the mothers were visiting the health facility during the first three months of their pregnancy. This was higher to other studies that 12.4% [14] and 20.5% [13] of the mothers had their first ANC visit during the first trimester. This proportion was also higher than the national (26.6%) and urban averages (39.1%) [12] and 25.5% of an Ethiopian study [15]. This higher level of first visit during the first trimester could be mainly due to the Ministry of Health effort on awareness of the mothers, and they were from urban community which could have better level of knowledge on early booking of antenatal care.

This study indicated that 84.1% of the mothers had good comprehensive knowledge. This was almost similar to a study in Benghazi, Libya (85.3%) [16], and higher to a study done in Maharashtra (58%) [4] and in Nigeria (44.2%) [1]. This higher level of knowledge reflects the interventions already done by the Ministry of Health and the reproductive health department in increasing awareness on different aspects of antenatal care by mass media, posters, and health professionals.

Almost all mothers had positive attitude on antenatal care, which was similar to a study in Benghazi, Libya (96.0%) [16], and higher to a study in Nigeria (53.8%) [1]. Even though pregnant mothers in Massawa subzone were having higher level of attitude and knowledge, they had lower level of practice (45%) on antenatal care. This level of practice was lower to a study in Maharashtra (69.3%) [4] and Libya (76.4%) [16]. This showed that their good knowledge and attitude cannot be translated to good practice due to many constraints.

Primigravid and nulliparous mothers had poor level of knowledge and practice compared to the multigravida and multiparous mothers. This further strengthened that the single and unemployed with age less than 20 years were having poor level of knowledge when compared to their counterparts. This displayed that being aged and having higher experience in antenatal care services and social and financial characteristics could have positive impact in their level of knowledge and practice on antenatal care.

This study revealed that age, marital status, occupation, and ethnicity had showed significant association to their comprehensive knowledge. Moreover; their level of gravidity and parity had indicated statistically significant association to their knowledge and practice. This was different to a study conducted in Pakistan that knowledge about antenatal care was much higher in primigravid, younger educated women [5]. This had shown that being multiparous, multigravida, married, and housewife have an option to have direct contact with health professionals on antenatal care visits for counseling.

## 5. Limitation of the Study

The study was conducted in a city and in a small study area, which the results cannot generalize the rural areas and the country as a whole. Taking larger sample and from different communities and subzones could have better result to generalize to the whole country. In addition to the interview, having practical observation to their practice was not done. It was a hospital-based study; thus, the mothers may had respondent's bias on the interview.

## 6. Conclusion

The mothers in Amatere Health Center had good level of knowledge and attitude, but they had lower level of practice on antenatal care. Multiparous and multigravida mothers were having higher level of knowledge and practice on antenatal care. Age, occupation, marital status, and ethnicity showed statistically significant association to their level of comprehensive knowledge. Most mothers favor hospital delivery, and the practice of starting antenatal care in the first three months was satisfactory. Even though it was small in number, underage marriage was seen in some mothers and few mothers had smoking and alcohol intake practice during their pregnancy.

## 7. Recommendations

Health professionals should advocate in increasing community awareness on antenatal care and the possible complications during delivery to decrease the preventable maternal deaths. Even though it is already started in some cities of the country, waiting homes should be strongly recommended to decrease home deliveries and neonatal and maternal complications. As the WHO recommendation, starting antenatal care as early as possible in the first trimester and having regular visits to about eight times should be advocated. Underage marriage should be discouraged, and mothers should be counseled to stop the harmful habits of smoking and alcohol consumption, which can predispose their baby to potential teratogenicity. Further national studies which include larger sample size, different study area, and ethnicity are highly recommended.

## Figures and Tables

**Table 1:** Sociodemographic characteristics of pregnant women in Amatere Health Center, Massawa, Eritrea.

Variables	Categories	Frequency (*N*)	Percent (%)
Age of respondents	<20	20	6.9
	20-29	171	59.2
	30-39	98	33.9

Marital status	Married	271	93.8
	Single	17	5.9
	Divorced	1	0.3

Ethnicity	Tigrigna	70	24.2
	Tigre	151	52.2
	Afar	17	5.9
	Saho	30	10.4
	Other	21	7.3

Religion	Christian	72	24.9
	Muslim	217	75.1

Educational level of respondent	No formal education	61	21.1
	Primary	75	28.0
	Middle	84	29.1
	High school	51	17.6
	Tertiary	18	6.2

Husband's education	No formal education	56	20.1
	Primary	35	12.5
	Middle	68	24.4
	High school	75	26.9
	Tertiary	45	16.1

Occupation	Housewife	223	77.2
	Self-employed	17	5.9
	Governmental	31	10.7
	Single-unemployed	18	6.2

Gravidity	One	60	20.8
	Two-four	150	52.1
	>Four	78	27.6

Parity	Zero	64	22.2
	One-four	197	68.4
	>Four	27	9.4

Total		289	100.0

**Table 2:** Knowledge of pregnant women on antenatal care in Amatere Health Center, Massawa, Eritrea.

Variables	Categories	Frequency (*N*)	Percent (%)
Place of delivery in previous pregnancy	Hospital	203	70.2
	Home	22	7.6
	Primigravid mother	64	22.1

When should the first antenatal checkup be started?	First 3 months	189	65.4
	After 3 months	90	31.1
	At delivery	1	0.3
	Do not know	9	3.1

Does a pregnant woman need to come for at least four antenatal checks throughout her pregnancy?	Yes	277	95.8
	No	1	0.3
	Cannot say	11	3.8

Is maternal smoking harmful to the fetus?	Yes	282	97.6
	No	2	0.7
	Do not know	5	1.7

Can alcohol consumption during pregnancy affect the fetal growth?	Yes	281	97.2
	No	3	1.0
	Do not know	5	1.7

Are you aware that any infection during pregnancy can cause harm to your baby?	Yes	268	92.7
	No	2	0.7
	Do not know	19	6.6

Any medicines other than those prescribed by doctor can cause harm to your baby?	Yes	284	98.3
	No	3	1.0
	Do not know	2	0.7

In your opinion, where should a pregnant woman deliver her baby?	Health facility	286	99.0
	Home	3	1.0

What are the danger signs of pregnancy?	Convulsion	206	71.3
	Vaginal bleeding	265	91.7
	Excessive vomiting	226	78.2
	Persistent swelling	228	78.9
	Weak fetal movement	233	80.6

What should be done in case of any such problem?	Report health center	288	99.7
	Home remedies	1	0.3

Total		289	100.0

**Table 3:** Attitude of pregnant women on antenatal care in Amatere Health Center, Massawa, Eritrea.

Variables	Responses *N* (%)				
	Strongly agree	Agree	Neutral	Disagree	Strongly disagree
Antenatal checkup is necessary	270 (93.4)	18 (6.2)	1 (0.3)	0 (0.0)	0 (0.0)
ANC follow-up is good	271 (93.8)	14 (4.8)	4 (1.4)	0 (0.0)	0 (0.0)
Antenatal booking should be done	246 (85.1)	15 (5.2)	14 (4.8)	12 (4.2)	2 (0.7)
Screening for infections (HIV, HBV) should ANC checkup	261 (90.3)	21 (7.3)	6 (2.1)	1 (0.3)	0 (0.0)
Blood pressure should be checked	261 (90.3)	22 (7.6)	6 (2.1)	0 (0.0)	0 (0.0)
USG as advised by doctor to monitor fetal growth	265 (91.7)	18 (6.2)	5 (1.7)	0 (0.0)	1 (0.3)
Pregnant women should change dietary habit as advised by doctor	265 (91.7)	16 (5.5)	7 (2.4)	1 (0.3)	0 (0.0)
Supplying of iron and folic acid is good for the mother and fetus	267 (92.4)	8 (2.8)	13 (4.5)	1 (0.3)	0 (0.0)
Home delivery is better than hospital delivery	11 (3.8)	1 (0.3)	4 (1.4)	36 (12.5)	237 (82.0)
Smoking does not cause harm to fetus	6 (2.1)	0 (0.0)	6 (2.1)	37 (12.8)	240 (83.0)
Alcohol consumption during pregnancy is good for fetus	5 (1.7)	0 (0.0)	6 (2.1)	39 (13.5)	239 (82.7)

**Table 4:** Practice of pregnant mothers on antenatal care in Amatere Health Center, Massawa, Eritrea (*N* = 289).

Variables	Categories	Frequency (*N*)	Percent (%)
At what duration of pregnancy did you visit the health center for ANC checkup?	First 3 months	170	59.4
	3-4 months	110	38.5
	At delivery	1	0.3
	Other	5	1.7

Are you regular in your antenatal visits for checkup?	Yes	275	96.8
	No	9	3.2

Number of ANC visit you have attended	One	4	1.5
	Two	9	3.3
	Three	18	6.7
	Four	70	26.0
	Five	117	43.5
	More than five	51	19.0

Are you taking iron and folic acid tablets?	Yes	250	88.7
	No	32	11.3

Did you ever smoke during pregnancy?	Yes	6	2.1
	No	281	97.9

Did you ever consume alcohol during pregnancy?	Yes	7	2.4
	No	280	97.6

Did you ever take medicine without consulting doctor?	Yes	10	3.5
	No	277	96.5

Did you suffer from any health problem on your current pregnancy?	Yes	30	10.5
	No	257	89.5

**Table 5:** Association between background of pregnant women and level of knowledge and attitude in Amatere Health Center, Massawa.

Variables	Comprehensive knowledge		*p* value	Comprehensive attitude		*p* value
Responses	Good *N* (%)	Poor *N* (%)		Good *N* (%)	Poor *N* (%)	
Age (years)						
<20	13 (65.0)	7 (35.0)	0.011	19 (95.0)	1 (5.0)	0.128
20-29	141 (82.5)	30 (17.5)		169 (98.8)	2 (1.2)	
30-39	89 (90.8)	9 (9.2)		98 (100.0)	0 (0.0)	
Marital status						
Married	234 (86.3)	37 (13.7)	0.001	269 (99.3)	2 (0.7)	0.127
Single	8 (47.1)	9 (52.9)		16 (94.1)	1 (5.9)	
Divorced	1 (100.0)	0 (0.0)		1 (100.0)	0 (0.0)	
Ethnicity						
Tigrigna	64 (91.4)	6 (8.6)	0.014	69 (98.6)	1 (1.4)	0.119
Afar	12 (70.6)	5 (29.4)		16 (94.1)	1 (5.9)	
Tigre	130 (86.1)	21 (13.9)		151 (100.0)	0 (0.0)	
Saho	20 (66.7)	10 (33.3)		29 (96.7)	1 (3.3)	
Other	17 (81.0)	4 (19.0)		21 (100.0)	0 (0.0)	
Religion						
Christian	65 (90.3)	7 (9.7)	0.097	71 (98.6)	1 (1.4)	0.735
Muslim	178 (82.0)	39 (18.0)		215 (99.1)	2 (0.9)	
Occupation						
Housewife	192 (86.1)	31 (13.9)	0.001	222 (99.6)	1 (0.4)	0.114
Self-employed	16 (94.1)	1 (5.9)		17 (100.0)	0 (0.0)	
Governmental	28 (90.3)	3 (9.7)		30 (96.8)	1 (3.2)	
Single-unemployed	7 (38.9)	11 (61.1)		17 (94.4)	1 (5.6)	
Level of education of respondent						
No formal education	49 (80.3)	12 (19.7)	0.609	61 (100.0)	0 (0.0)	0.305
Primary	65 (86.7)	10 (13.3)		74 (98.7)	1 (1.3)	
Middle	69 (82.1)	15 (17.9)		83 (98.8)	1 (1.2)	
High school	43 (84.3)	8 (15.7)		51 (100.0)	0 (0.0)	
Tertiary	17 (94.4)	1 (5.6)		17 (94.4)	1 (5.6)	
Husband's education						
No formal education	48 (85.7)	8 (14.3)	0.357	56 (100.0)	0 (0.0)	0.558
Primary	26 (74.3)	9 (25.7)		34 (97.1)	1 (2.9)	
Middle	56 (82.4)	12 (17.6)		68 (100.0)	0 (0.0)	
High school	67 (89.3)	8 (10.7)		74 (98.7)	1 (1.3)	
Tertiary	37 (82.2)	8 (17.8)		44 (97.8)	1 (2.2)	
Gravid						
One	29 (48.3)	31 (51.7)	0.001	57 (95.0)	3 (5.0)	0.174
Two-four	140 (93.3)	10 (6.7)		150 (100.0)	0 (0.0)	
>Four	73 (93.6)	5 (6.4)		78 (100.0)	0 (0.0)	
Parity						
Zero	32 (50.0)	32 (50.0)	0.001	61 (95.3)	3 (4.7)	0.157
One-four	187 (94.9)	10 (5.1)		197 (100.0)	0 (0.0)	
>Four	23 (85.1)	4 (14.9)		27 (100.0)	0 (0.0)	
Total	243 (84.1)	46 (15.9)		285 (99.0)	3 (1.0)	

**Table 6:** Association between background characteristics and comprehensive practice of pregnant women in Amatere Health Center, 2019.

Variables	Categories	Comprehensive practice				*p* value
		Good practice		Poor practice		
		Frequency	Percent	Frequency	Percent	
Age (years)	<20	6	30.0	14	70.0	0.083
	20-29	72	42.1	99	57.9	
	30-39	52	53.1	46	46.9	

Marital status	Married	124	45.8	147	54.2	0.228
	Single	5	29.4	12	70.6	
	Divorced	1	100.0	0	0.0	

Ethnicity	Tigrigna	39	55.7	31	44.3	0.138
	Afar	4	23.5	13	76.5	
	Tigre	66	43.7	85	56.3	
	Saho	13	43.3	17	56.7	
	Other	8	38.1	13	61.9	

Religion	Christian	40	55.6	32	44.4	0.037
	Muslim	90	41.5	127	58.5	

Occupation	Housewife	99	44.4	124	55.6	0.479
	Self-employed	10	58.8	7	41.2	
	Governmental	15	48.4	16	51.6	
	Single-unemployed	6	33.3	12	66.7	

Level of education of respondent	No formal education	25	41.0	36	59.0	0.872
	Primary	32	42.7	43	57.3	
	Middle	41	48.8	43	51.2	
	High school	23	45.1	28	54.9	
	Tertiary	9	50.0	9	50.0	

Husband's education	No formal education	21	37.5	35	62.5	0.469
	Primary	14	40.0	21	60.0	
	Middle	34	50.0	34	50.0	
	High school	35	46.7	40	53.3	
	Tertiary	24	53.3	21	46.7	

Gravid	One	13	21.7	47	78.3	0.003
	Two-four	75	50.0	75	50.0	
	>Four	33	47.9	36	52.1	

Parity	Zero	13	20.3	51	79.7	0.001
	One-four	107	54.4	90	45.6	
	>Four	10	37.1	17	62.9	

Total		130	45.1	158	54.9	

## Data Availability

Availability of additional supplementary materials can be requested from the corresponding author as needed.
